# The Bony Density of the Pedicle Plays a More Significant Role in the Screw Anchorage Ability Than Other Regions of the Screw Trajectory

**DOI:** 10.1111/os.14299

**Published:** 2024-11-22

**Authors:** Zan Chen, Yue Chen, Jiajun Zhou, Yanwei He, Jingchi Li

**Affiliations:** ^1^ Department of Orthopedic The Affliated Hospital of Southwest Medical University Luzhou China; ^2^ Nuclear Medicine and Molecular Imaging Key Laboratory of Sichuan Province The Affiliated Hospital of Southwest Medical University Luzhou China; ^3^ Southwest Medical University Luzhou China; ^4^ Department of Sports Medicine Huashan Hospital, Fudan University Shanghai China; ^5^ Luzhou Key Laboratory of Orthopedic Disorders, Department of Orthopedics The Affiliated Traditional Chinese Medicine Hospital of Southwest Medical University Luzhou China

**Keywords:** fixation strength computation, Hounsfield‐unit value, pedicle, screw anchorage ability, screw loosening

## Abstract

**Objective:**

Osteoporosis is a crucial risk factor for screw loosening. Our studies indicate that the bone mineral density (BMD) in the screw trajectory is a better predictor of screw loosening than the BMD of the lumbar spine or the screw insertion position. Research has shown that anchorage on the screw tip is the most significant factor for screw anchorage ability, while others argue that decreased bony quality in the pedicle poses a significant risk for screw loosening. This study aimed to determine whether the bony quality of the screw tip, pedicle, or screw‐anchored vertebral body plays the most significant role in screw anchorage ability.

**Methods:**

A total of 73 patients who underwent single–segment bilateral pedicle screw fixation, along with posterior and transforaminal lumbar interbody fusion (PLIF and TLIF), from March 2019 to September 2020 were included in this retrospective study. The Hounsfield unit (HU) value of the fixed vertebral bodies, the entire screw trajectory, screw tip, screw–anchoraged vertebral body, and pedicles were measured separately. Data from patients with and without screw loosening were compared, and regression analyses were conducted to identify independent risk factors. Additionally, the area under the curve (AUC) values were computed to assess the predictive performance of different parameters. Furthermore, fixation strength was calculated in numerical models with varying bony densities in different regions.

**Results:**

HU values were found to be significantly lower in the loosening group across most measuring methods (HU values in the pedicle, 148.79 ± 97.04, 33.06 ± 34.82, *p* < 0.001). Specifically, the AUC of screw loosening prediction was notably higher when using HU values of the pedicle compared to other methods (AUC in the pedicle > 0.9 and in the screw insertion position > 0.7). Additionally, computational results for fixation strength revealed a clear decline in screw anchorage ability in models with poor BMD in the pedicle region.

**Conclusions:**

Pedicle bone quality plays a more significant role in screw anchorage ability than that in other regions. The innovation of bony augmentation strategies should pay more attention to this region to optimize the screw anchorage ability effectively.

## Graphical abstract

Pedicle bony density predicts risk of pedicle screw loosening.

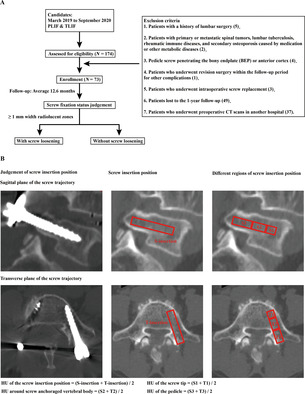

## 1 Introduction

Bilateral pedicle screw fixation is a standard surgical method during the construction of segmental stability in spine surgery [1, 2]. As a hardware‐related complication, screw loosening is common, which negatively affects fixation stability in surgical segments, triggers several postoperative complications (e.g., nonunion and pseudoarthrosis), and deteriorates the patient's long‐term outcomes [3, 4]. Biomechanical deterioration is the main trigger for screw loosening. Therefore, biomechanical changes should reasonably explain screw loosening‐related clinical factors and provide theoretical references for optimizing treatment strategies [5, 6]. Osteoporosis is common, especially in elderly patients [7, 8]. Osteoporosis causes the loss of bony strength, and microfractures can occur under the same size load in osteoporotic vertebral bodies [9, 10]. Moreover, osteoporosis can also aggravate the stiffness difference between the screw and bony structure, and the corresponding stress concentration on the bone–screw interface is another significant reason for screw loosening [11, 12]. Therefore, the judgment of patients' bony density is necessary to evaluate the potential risk of screw loosening for these patients.

Traditionally, dual–energy X‐ray absorptiometry (DXA) is the gold standard for detection. However, new formation, such as osteophytes, may influence the results of the *T* score, leading to underestimation of cancellous bony density reduction and screw loosening incidence [13, 14]. In contrast, the vertebral Hounsfield unit (HU) value on preoperative computed tomography (CT) is more credible when evaluating the bone mineral density (BMD) of the vertebral body, and the risk of screw loosening for adjusting the region of interest (ROI) could effectively eliminate the confounding effect caused by osteophytes [15, 16]. Moreover, significant regional variations in BMD are observed within the vertebral body. It is the osteoporosis–affecting local cancellous microstructures, rather than the entire vertebral body, that contributes to the incidence of screw loosening [17, 18]. Consequently, when utilizing HU from the vertebral body to represent BMD along the screw trajectory, it is essential to acknowledge that confounding effects arising from regional differences in BMD cannot be disregarded. Correspondingly, our published studies show that HU values measured in the screw trajectory can better predict screw loosening risk than the vertebral body HU [18, 19]. From the biomechanical perspective, the trajectory of the pedicle screw can be divided into different regions, including bony structures around the screw tip, on the screw anchorage centrum region, and the pedicle. Studies have shown that anchorage at the screw tip is the most important factor for screw anchorage ability [20, 21], but others believe that a reduction in the bone quality of the pedicle is a significant risk factor for screw loosening [22, 23]. In other words, whether bony quality plays the most significant role in screw anchorage ability has yet to be identified. Identifying this topic could provide theoretical guidance for the selection and optimization of screw trajectory augmentation strategies. The aim of this study is to investigate the following three aspects: (i) to analyze the HU values in different regions of the screw trajectory; (ii) to compare which screw trajectory region has the most substantial impact on screw anchorage ability; and (iii) to assess screw anchorage capacity using numerical mechanical simulation.

## 2 Materials and Methods

### 2.1 Clinical Review

#### 2.1.1 Patient Data Collection

The current study protocol received approval from the ethics committees of our hospital (KY2023291). Informed consent was waived for this retrospective study. We conducted a retrospective review of the demographic and radiographic data of 73 patients who underwent bilateral pedicle screw fixed posterior and transforaminal lumbar interbody fusion (PLIF and TLIF) in the L4–L5 motion segment from March 2019 to September 2020. The average follow‐up period for these patients was 12.6 months (ranging from 11.4 to 14.2 months). The PLIF and TLIF procedures were performed by a senior spine surgeon, using only pedicle screws with lengths of 60 and 65 mm and diameters of 4.0 and 4.5 mm, which were identical on the left and right sides.

The selection of screw diameter and length was based on the dimensions of the pedicle and the size of the patients' vertebral bodies. The average height of the current patient cohort was 163 cm, which corresponds to relatively smaller vertebral body sizes in these individuals. Screw thread types (single or dual thread) were recorded according to the medical record and imaging data. All screws were placed in a single attempt without penetrating the anterior cortex. To ensure complete screw insertion, we removed the cortical shell surrounding the insertion point in the current patient series.

The inclusion criteria were as follows: (1) patients who underwent bilateral pedicle screw fixed PLIF and TLIF for lumbar degenerative diseases, including lumbar disc herniation, spinal stenosis, and spondylolisthesis; (2) patients underwent lumbar CT two times, including 1 week before and after the operation; and (3) patients underwent anterior–posterior and lateral radiography in the 1‐year follow‐up period. The exclusion criteria were as follows: (1) patients with a history of lumbar surgery; (2) patients with primary or metastatic spinal tumors, lumbar tuberculosis, rheumatic immune diseases, and secondary osteoporosis caused by medication or other metabolic diseases; (3) pedicle screw penetrating the bony endplate (BEP) or anterior cortex; (4) patients who underwent lumbar revision surgery within the clinical follow‐up period of 12 months for complications other than screw loosening; (5) patients who underwent intraoperative screw replacement; (6) patients lost to the 1‐year follow‐up; and (7) patients who underwent preoperative CT scans in another hospital.

#### 2.1.2 Assessment of Screw Loosening and HU Measured by Different Methods

The screw loosening status of the L5 vertebral body was judged based on 1 year of postoperative anterior–posterior and lateral radiography. Screw trajectories with ≥ 1 mm width radiolucent zones around the screw were defined as screw loosening [14, 24]. All patients underwent lumbar CT scans two times in our hospital's radiology department: once 1 week before the operation and once 1 week after. The tube voltage was set as 120 kV, and this parameter was identical to studies with the same topic [17, 19]. Imaging data from different CT scans play different roles in this study. All HU value measurement procedures were performed in the preoperative CT scan. During the measurement of the vertebral body HU, the ROI was placed in four planes: the midsagittal plane, central transverse plane, transverse plane close to the superior plane, and inferior endplate. Cancellous bone included in the ROI, cortical bone, bony endplates, posterior structure osteophytes, and posterior venous plexus were excluded [25, 26].

**FIGURE 1:**
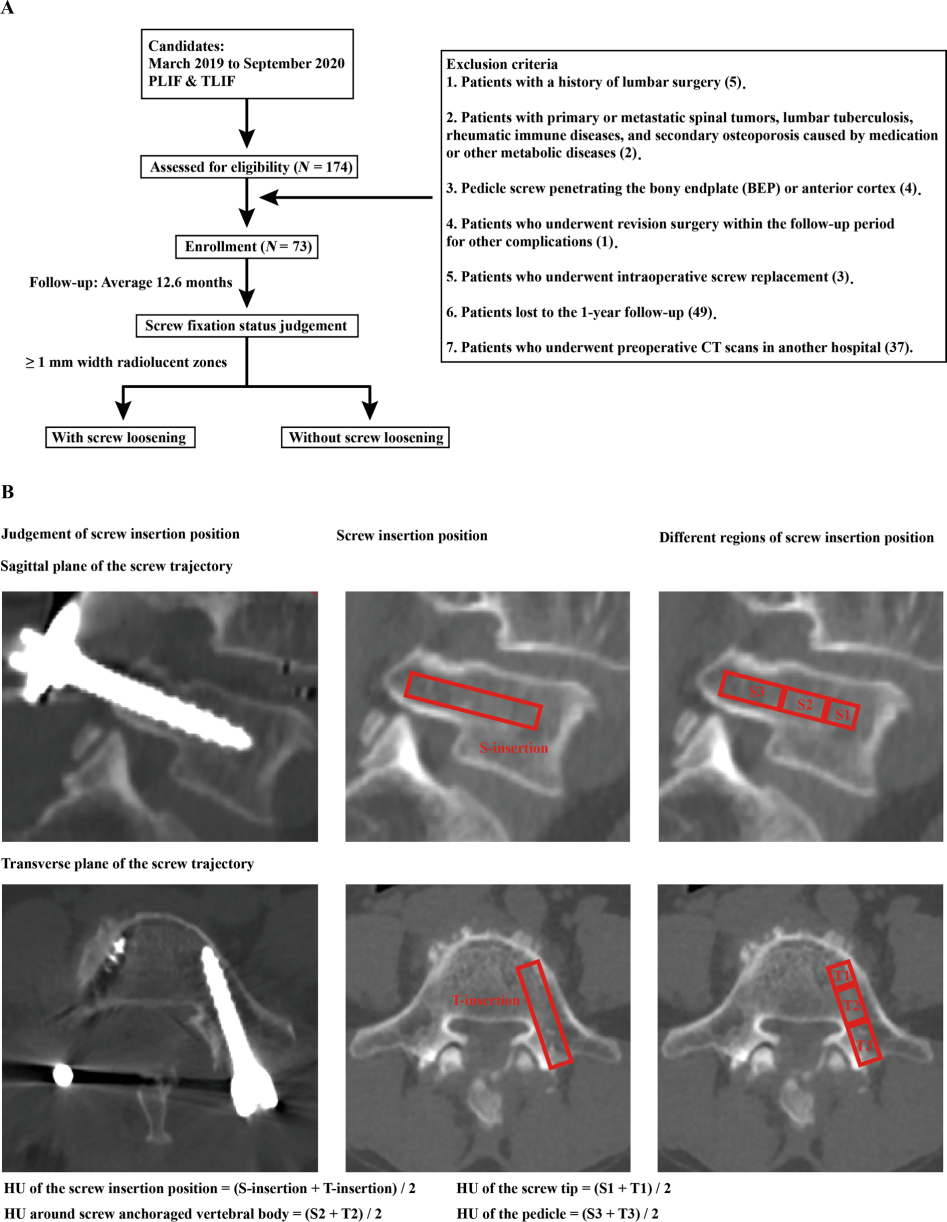
(A) Schematic for patients inclusion and exclusion criteria. (B) Illustration of measurement of HU values in the screw insertion position and different regions of the screw insertion position.

It is worth noting that the bony density of the cancellous bone along the screw trajectory provides the primary anchorage for pedicle screws. The measurement of HU values exclusively within cancellous bone is a commonly employed method in research addressing risks associated with screw loosening [14, 22]. The average HU values of these four planes were computed to represent the HU of the vertebral body [16, 17]. When evaluating HU values in the screw trajectory, the trajectory of pedicle screws in the preoperative CT was judged according to the instant postoperative CT scan, and the HU value of the screw trajectory was measured in the central sagittal plane of the screw (rather than the vertebral body's sagittal plane) [18, 19]. When evaluating BMD in different regions of the screw trajectory, HU values were separately measured in the screw tip (approximately 1–2 threads on the screw tip), screw–anchoraged vertebral body, and pedicle (Figure 1).

#### 2.1.3 Statistical Analyses

Statistical analyses were conducted using SPSS 23.0 software (IBM, USA) and R software (Version 4.1.1). To judge the interobserver and intraobserver reliability, 10 patients were randomly selected. One week after the measurement of these imaging data, the imaging data of these selected patients were remeasured by the radiologist and a well‐trained spine surgeon. The intraclass correlation efficiency (ICC) was computed to identify the repeatability of measured HU values (ICC ≥ 0.8 represents excellent reliability) [17, 27]. The *κ* values were computed to determine the interobserver and intraobserver repeatability during the judgment of screw loosening (*κ* values of 0.41–0.60 indicated moderate reliability; 0.61–0.80 indicated substantial agreement; and 0.81–1.00 indicated excellent or almost perfect agreement) [28, 29].

**FIGURE 2:**
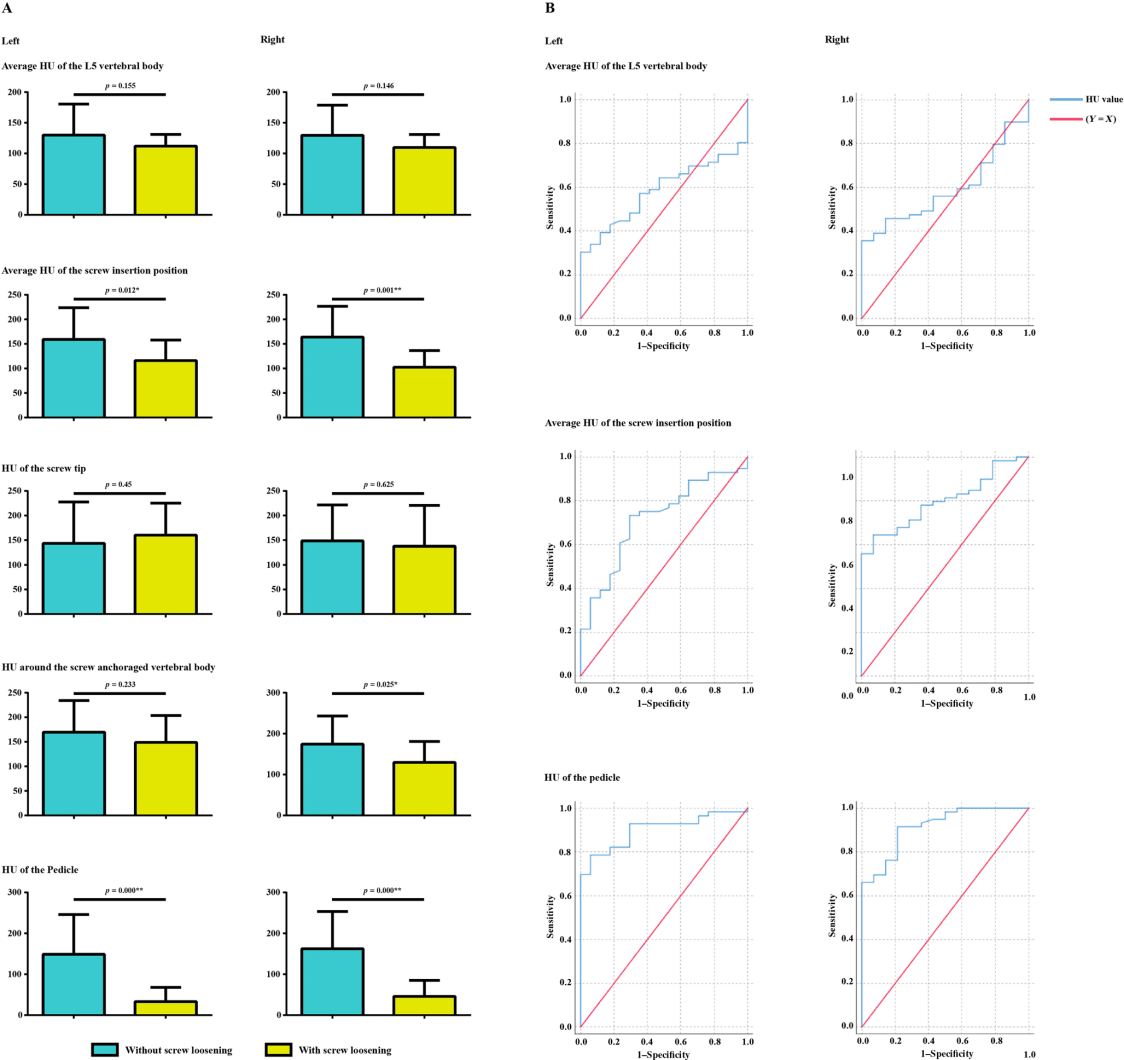
(A) Significant differences in HU values in groups with and with screw loosening. (B) ROC curves for screw loosening prediction: The predictive performance of screw insertion position's HU value was better than the average value of vertebral body, and that of the pedicle was better than the screw insertion position.

**FIGURE 3:**
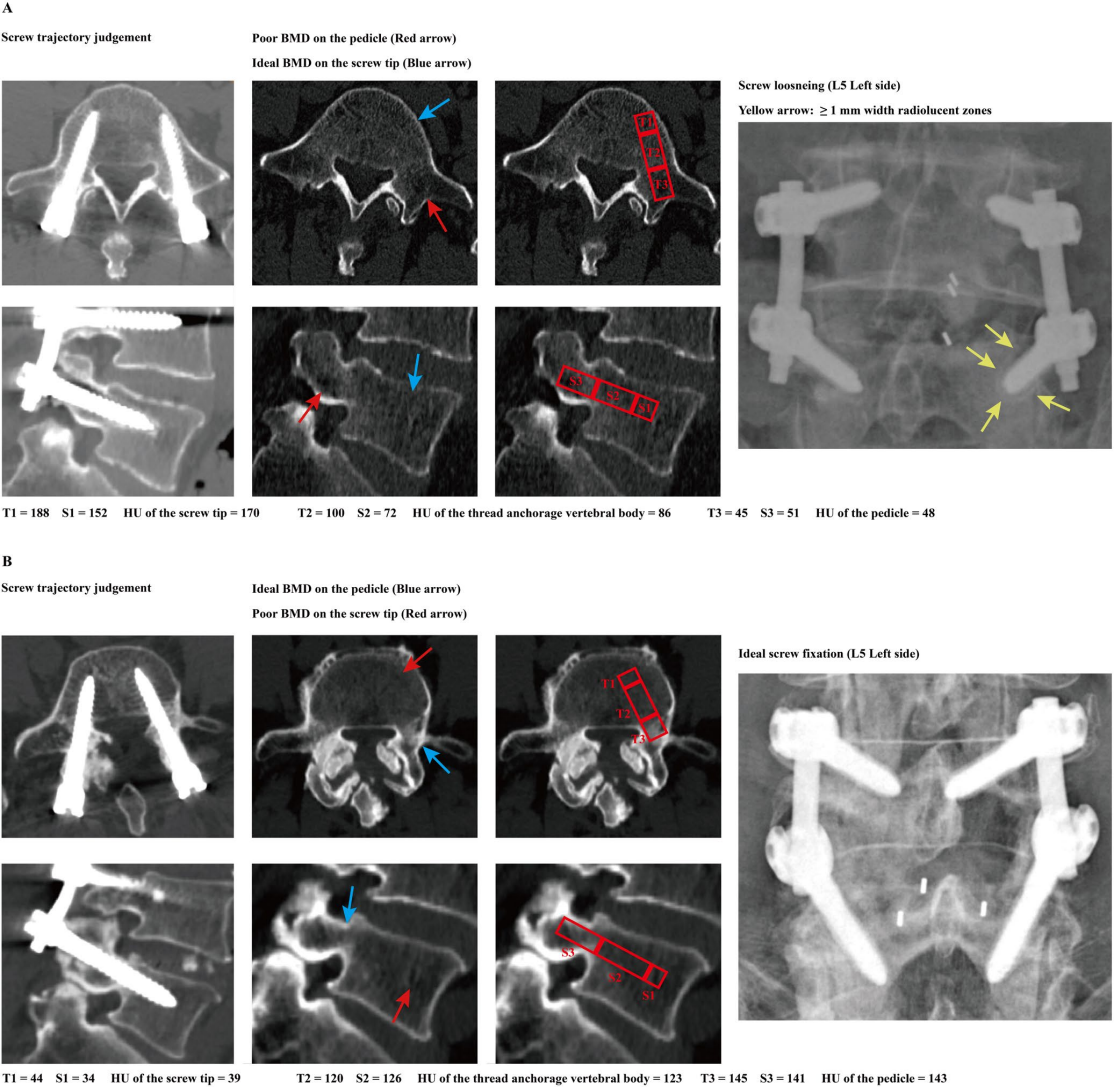
In cases of patients with varying regional BMD at the screw insertion site and differing clinical outcomes (with or without screw loosening) during a 1–year follow‐up, the predominant role of pedicle region's BMD in predicting screw loosening is clearly evident. (A) A typical case with ideal BMD in the screw tip (HU = 170), poor BMD in the pedicle (HU = 48), and with screw loosening in 1–year follow‐up. (B) A typical case with poor BMD in the screw tip (HU = 39), ideal BMD in the pedicle (HU = 143), and without screw loosening in 1–year follow‐up.

When comparing the difference between screw loosening status (nonloosening and loosening). The normality test was performed for all HU values. The independent samples Student's *t*‐test was used for HU values measured by different methods because these indicators are normally distributed [18, 30]. A *p* value less than 0.05 indicated a significant difference. We performed binary logistic regression to identify independent risk factors for screw loosening [17, 19]. Because excellent consistency between HU values measured by different methods existed (according to the computation of ICC), HU values were included in the regression analyses separately [31, 32]. Variables with *p* < 0.05 were considered independent risk factors in the multivariate analyses. Univariate analyses of each potential risk factor were performed, and the variables that achieved a significance level of *p* < 0.1 were entered into multivariate analyses. If only *p* values of HU measured by different methods were *p* < 0.1 (i.e., no *p* values of demographic covariates were < 0.1), we judged whether HU values were independent risk factors based on univariate logistic regression analysis results. Finally, we performed ROC curve analyses to assess the predictive value of HU measured by different methods, and the area under the curve (AUC) was calculated as an indicator to judge the predictive performance [33, 34]. Significant differences in AUC were compared between HU measurement methods (Figures 1, 2, 3).

### 2.2 Fixation Strength Computation in Numerical Models With Different Screw Trajectory BMD

#### 2.2.1 Model Construction

**FIGURE 4:**
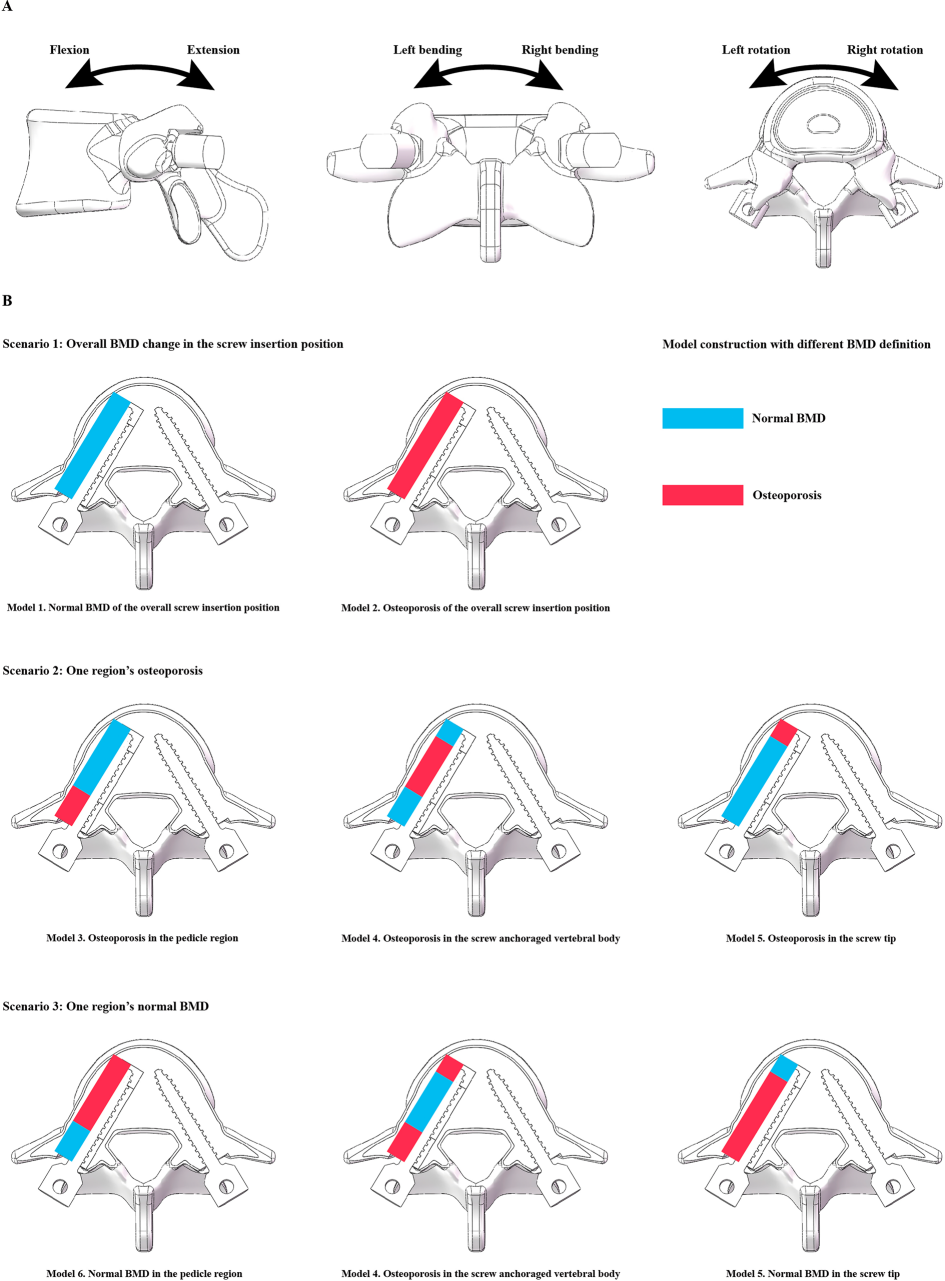
(A) Schematics outlining the L5 vertebral body, as well as the boundaries and loading conditions for computing fixation strength. (B) Construction scenarios for models with varying bone mineral density (BMD) in different regions of the screw insertion position: Scenario 1: Two models with different BMD along the screw trajectory.. Scenario 2: Three different models with osteoporosis in one region.. Scenario 3: Three different models with normal BMD in one region..

The L5 vertebral body was selected to simulate the fixation strength of a single vertebral body. The model construction strategy has been widely reported in our published studies. Specifically, bone structures include cortical, cancellous, and bony endplates (BEPs). The cortical thickness was set as 0.8 mm, and the thickness and morphology parameters (i.e., concave angles and depths) of BEPs were defined separately based on anatomic and imaging studies [35, 36, 37]. When simulating pedicle screw fixation, bilateral pedicle screws were inserted into the L5 vertebral body (diameter: 6.5 mm; length: 40 mm). The axes of the screws in the transverse plane were parallel to the pedicle axis, and those in the sagittal plane were parallel to the superior BEPs [38, 39]. The BMD of bony structures was defined as osteoporosis except for the left side screw insertion position (including the right one). When simulating BMD changes in different regions of the screw trajectory, the elastic modulus of the screw tip, screw anchoraged vertebral body, and pedicle were adjusted separately [18, 40]. Specifically, the right side screw trajectory with normal BMD and osteoporosis was simulated in Models 1 and 2. Models with osteoporosis in only one region of the screw trajectory were simulated in Models 3–5, and models with normal BMD in only one region were simulated in Models 6–8. By using this model construction strategy, the effect of BMD changes in different regions of the screw insertion position on the screw anchorage ability can be effectively discretized (Tables 1 and 2 and Figure 4).

**TABLE 1:** Material properties of FE models' components.

Components	Elastic modulus (MPa)	Poisson's ratio
Cortical (Osteoporosis)	E_xx_ = 7571 E_yy_ = 7571 E_zz_ = 14,740 G_xy_ = 2546 G_yz_ = 3618 G_xz_ = 3618	V_xy_ = 0.484 V_yz_ = 0.203 V_xz_ = 0.203
Cancellous (normal BMD)	E_xx_ = 140 E_yy_ = 140 E_zz_ = 200 G_xy_ = 48.3 G_yz_ = 48.3 G_xz_ = 48.3	V_xy_ = 0.45 V_yz_ = 0.315 V_xz_ = 0.315
Cancellous (osteoporosis)	E_xx_ = 47.6 E_yy_ = 47.6 E_zz_ = 100 G_xy_ = 16.42 G_yz_ = 24.15 G_xz_ = 24.15	V_xy_ = 0.45 V_yz_ = 0.315 V_xz_ = 0.315
Bony endplates (osteoporosis)	8070	0.3
Titanium alloy screw	120,000	0.31

**TABLE 2:** Model construction strategy for left‐side screw trajectory in vertebral bodies with different regional BMD.

	Screw tip	Screw anchoraged vertebral body	Pedicle
Scenario 1: Overall BMD change in the screw insertion position			
Model 1	+	+	+
Model 2	−	−	−
Scenario 2: One region's osteoporosis			
Model 3	+	+	−
Model 4	+	−	+
Model 5	−	+	+
Scenario 3: One region's normal BMD			
Model 3	−	−	+
Model 4	−	+	−
Model 5	+	−	−

*Note*: +, Normal BMD; −, osteoporosis.

#### 2.2.2 Boundary and Loading Conditions

Interfaces between the vertebral body and screws were defined as friction contact, and the frictional coefficient was set as 0.2 [41, 42]. The freedom–degree screw tulip was completely fixed, and different directional moments, including flexion, extension, bending, and rotation, were applied on the superior surface of the vertebral body [43, 44]. We performed a mesh convergence test on the L5 vertebral body by evaluating the change in the maximum stress of the pedicle screw under the 10 Nm flexion loading condition. The model was considered converged if the change in the computed stress value was < 3% [45, 46]. Displacement and stress values under 10 Nm different directional moments (including flexion, extension, and left and right bending and rotation) were recorded separately to judge screw anchorage ability in different models.

## 3 Results

### 3.1 Retrospective Review of the Clinical Data

#### 3.1.1 Patient Data Collection and Significant Difference Verification

A total of 73 patients who underwent L4–L5 PLIF and TLIF with bilateral pedicle screw fixation were included in this study. The overall incidence rates of screw loosening were 23.29% and 19.18% on the left and right sides, respectively, with no significant difference between the two sides (*p* = 0.295). The interobserver and intraobserver reliability was excellent, as evidenced by the ICC values of HU measurement (0.846 and 0.911, respectively) and *κ* values (0.875 and 0.886, respectively). There were no significant differences in demographic covariates (age, sex, BMI, smoking history), and surgical method selection (PLIF or TLIF) between patients with or without screw loosening on either side. Moreover, while the incidence of screw loosening was numerically lower in patients with dual‐thread pedicle screw fixation, this difference was not statistically significant. Furthermore, this factor was not identified as an independent risk factor for screw loosening.

The differences in the L5 vertebral body HU and the HU of the screw tips were also insignificant between the two groups, and the HU value of the screw tip was insignificantly higher in patients with left‐side screw loosening. In contrast, the HU values of the screw–anchoraged vertebral body were significantly higher in patients without right‐side screw loosening but were still insignificantly higher in the left group. Moreover, the HU values of the screw insertion positions and pedicle regions were significantly higher in patients without screw loosening on both the left and right sides. The differences in HU values were largest in the pedicle region in patients with and without screw loosening (Table 3).

**TABLE 3:** Covariates for patients with and without L5 vertebral screw loosening.

	Without screw loosening	With screw loosening	*p*
Left side			
Demographic covariates			
Age	63.45 ± 7.7	60.82 ± 9.76	0.252
Sex (male/female)	24/32	9/8	0.464
BMI	24.69 ± 3.2	24.44 ± 3.13	0.774
Smoking history	49/7	14/3	0.589
HU values			
Screw tip	143.46 ± 84.04	160.35 ± 65.33	0.45
Thread around the vertebral body	169.63 ± 64.45	148.85 ± 54.76	0.233
Pedicle	148.79 ± 97.04	33.06 ± 34.82	0.000**
Screw insertion position	159.05 ± 64.64	115.88 ± 41.92	0.012*
Average HU of vertebral body	129.99 ± 50.45	111.98 ± 19.02	0.155
Surgical method selection (PLIF/TLIF)	29/27	11/6	0.349
Screw length (mm)	6.24 ± 0.25	6.27 ± 0.26	0.737
Screw diameter (mm)	4.25 ± 0.25	4.32 ± 0.29	0.294
Screw type (single/dual thread)	41/15	15/2	0.199
Right side			
Demographic covariates			
Age	61.97 ± 7.98	66.5 ± 8.53	0.063
Sex (male/female)	25/34	8/6	0.316
BMI	24.67 ± 3.03	24.46 ± 2.58	0.828
Smoking history	51/8	12/2	0.943
HU values			
Screw tip	148.45 ± 73.1	137.5 ± 83.3	0.625
Thread around the vertebral body	174.35 ± 68.68	129.71 ± 51.17	0.025*
Pedicle	162.26 ± 91.19	45.96 ± 39.19	0.000**
Screw insertion position	163.9 ± 62.74	102.43 ± 34.01	0.001**
Average HU of vertebral body	129.59 ± 49.09	109.81 ± 21.09	0.146
Surgical method selection (PLIF/TLIF)	34/25	6/8	0.318
Screw length (mm)	6.25 ± 0.25	6.25 ± 0.26	0.955
Screw diameter (mm)	4.25 ± 0.25	4.36 ± 0.23	0.137
Screw type (single/dual thread)	42/17	12/2	0.265

*Note*: *Statistical significance in the multivariate regression analysis (*p* < 0.05). **Statistical significance in the multivariate regression analysis (*p* < 0.01).

#### 3.1.2 Independent Risk Factors and Parameter Prediction Values for Screw Loosening

On the left side, given that the *p* values of all demographic covariates were > 0.1 in univariate logistic regression analyses, whether HU values measured by different methods weree *p* value of patient age, screw–anchoraged vertebral body, pedicle, and screw insertion position were < 0.1 in univariate logistic regression analyses; therefore, age and HU values measured by these three different methods were separately enrolled in the multivariate analysis, and the result was consistent with the left side: only lower HU values in the pedicle and the screw insertional position were independent risk factors for screw loosening (Tables 4 and 5). Finally, the AUC of HU values measured in the pedicle was > 0.9, while that measured in the screw insertion position was > 0.7. The predictive performance of the pedicle region's HU values was significantly better than that of all other parameters on both the left and right sides (Table 6).

**TABLE 4:** Logistic regression analysis of the left side screw loosening.

	OR	95% CI		*p*
Demographic covariates				
Age	0.959	0.894	1.03	0.251
Sex (male/female)	0.466	0.224	1.982	0.667
BMI	0.975	0.82	1.159	0.771
Smoking history	1.5	0.342	6.571	0.591
Surgical method selection (PLIF/TLIF)	1.707	0.554	5.254	0.351
Screw length (mm)	1.46	0.166	12.841	0.733
Screw diameter (mm)	3.36	0.355	31.839	0.291
Screw type (single/dual thread)	0.971	0.808	1.618	0.2
HU values				
Screw tip	1.003	0.996	1.009	0.446
Thread around the vertebral body	0.944	0.985	1.004	0.232
Pedicle	0.972	0.958	0.987	0.000**
Screw insertion position	0.986	0.975	0.997	0.016*
Average HU of vertebral body	0.99	0.976	1.004	0.159

*Note*: *Statistical significance (*p* < 0.05). **Statistical significance (*p* < 0.01).

**TABLE 5:** Logistic regression analysis of the left side screw loosening.

	OR	95% CI		*p*
Univariate analyses				
Demographic covariates				
Age	1.059	0.995	1.149	0.069^#^
Sex (male/female)	0.551	0.17	1.971	0.322
BMI	0.979	0.813	1.179	0.825
Smoking history	1.062	0.2	5.657	0.943
Surgical method selection (PLIF/TLIF)	0.551	0.17	1.791	0.322
Screw length (mm)	1.07	0.104	11.077	0.955
Screw diameter (mm)	6.688	0.531	84.283	0.142
Screw type (single/dual thread)	0.98	0.814	1.73	0.265
HU values				
Screw tip	0.998	0.99	1.006	0.62
Thread around the vertebral body	0.987	0.976	0.999	0.031^*^
Pedicle	0.964	0.946	0.983	0.000^**^
Screw insertion position	0.976	0.961	0.992	0.003^**^
Average HU of vertebral body	0.988	0.973	1.004	0.151
Multivariate analysis				
Age	1.041	0.964	1.125	0.301
Thread around the vertebral body	0.989	0.977	1.001	0.079^#^
Age	1.072	0.965	1.19	0.195
Pedicle	0.963	0.944	0.983	0.000**
Age	1.025	0.946	1.111	0.544
Screw insertion position	0.977	0.962	0.993	0.005**

*Note*: ^#^Variables that achieved a significance level of *p* < 0.1 in the univariate analysis. *Statistical significance (*p* < 0.05). **Statistical significance (*p* < 0.01).

**TABLE 6:** ROC analysis for screw loosening prediction.

	Cut‐off value	Sensitivity	Specificity	AUC
Left				
Screw tip	133.25	0.706	0.536	0.589
Thread around the vertebral body	151	0.536	0.706	0.591
Pedicle	71.25	0.786	0.941	0.9
Screw insertion position	124	0.732	0.806	0.716
Average HU of vertebral body	116.5	0.571	0.647	0.584
Right				
Screw tip	134.5	0.492	0.786	0.561
Thread around the vertebral body	146	0.407	0.786	0.694
Pedicle	67.25	0.915	0.786	0.916
Screw insertion position	124.25	0.712	0.714	0.798
Average HU of vertebral body	121.5	0.492	0.643	0.59

#### 3.1.3 Numerical Mechanical Simulations

**FIGURE 5:**
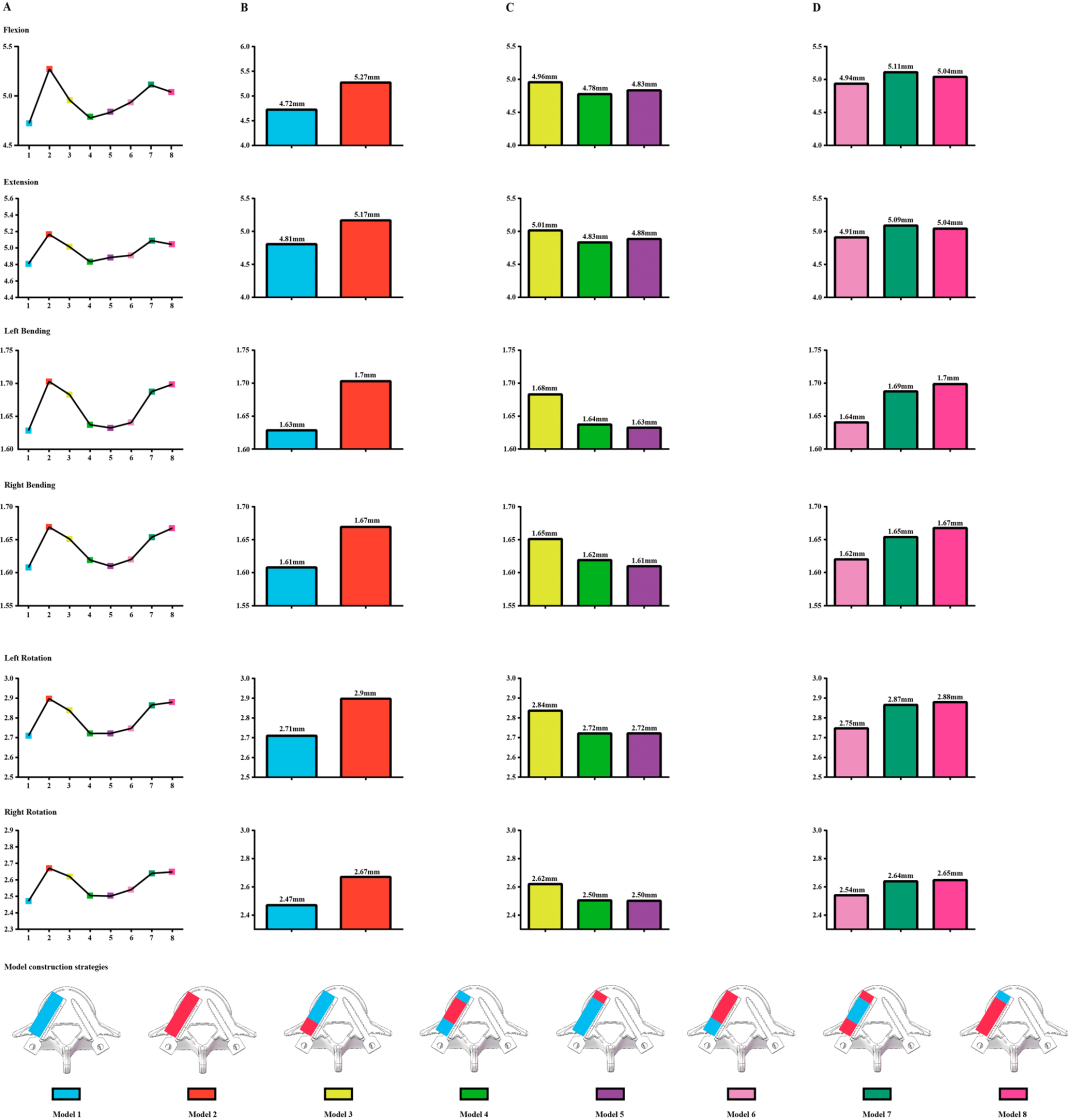
Computed screw displacement values in different models show that overall, better screw anchorage ability (i.e., lower screw displacement values) can be observed in models with normal BMD. In Scenario 1, a higher screw displacement value is observable in the model with an osteoporotic screw trajectory. In Scenario 2, the largest screw displacement value is recorded in the model with an osteoporotic pedicle. In Scenario 3, the lowest screw displacement value is recorded in the model with normal BMD in the pedicle. Therefore, biomechanical research validates the clinically observed phenomenon: Compared to other regions (i.e., screw tip and anchored vertebral body), BMD changes on the pedicle play a determinative role in screw anchorage ability. Changes in displacement values in (A) different models (mm); (B) Scenario 1 (mm); (B) Scenario 2 (mm); (C) Scenario 3 (mm).

Screw maximum displacement values were recorded in different models to judge changes in fixation stability. Consistent with published studies, considering that the stiffness of titanium alloy pedicle screws was dramatically higher than that of bony structures, the deformation of pedicle screws was ignored in this study. The computational results recorded the lowest screw displacement value in the model with the normal BMD of the overall screw insertion position, and the largest screw displacement value was also recorded in the overall osteoporosis model. In contrast, in models with only one region of osteoporosis, the largest screw displacement value can be observed in the model with pedicle region osteoporosis. Similarly, in models with only one region's ideal BMD, the lowest screw displacement value can be observed in the model with the pedicle region's normal BMD. This variation tendency was consistent under all directional loading conditions. Moreover, compared to the model with ideal BMD in the pedicle region and osteoporosis in the screw tip and screw–anchoraged vertebral body, a larger screw displacement value can be observed in models with an osteoporotic pedicle and ideal BMD in the other two regions under all loading conditions (Figure 5).

## 4 Discussion

### 4.1 Principal Findings

By conducting a comprehensive study that includes clinical reviews and corresponding biomechanical simulations, it has been determined that the HU value in the pedicle region plays a more significant role compared to other regions. The mechanism underlying this phenomenon has also been validated through numerical simulations. Specifically, density reduction in the pedicle can lead to a more pronounced deterioration of screw anchorage ability when compared to bony density reductions in other positions. Therefore, based on this research, augmentation techniques should place greater emphasis on the pedicle region, as this may serve as an alternative method to enhance screw anchorage ability and mitigate the associated risk of screw loosening, particularly in osteoporotic patients.

### 4.2 Background Review and Research Results

Local stress concentration serves as an initial trigger for the progression of aseptic screw loosening [47, 48]. Accordingly, comprehensive research comprising clinical reviews and biomechanical simulations can effectively assess the risk factors associated with aseptic screw loosening and elucidate its underlying biomechanical mechanisms. This approach is widely adopted in similar studies within this field [5, 18]. Osteoporosis is an important risk factor for screw loosening [18, 19]. Compared to the *T* score measured by DXA, the HU value could better evaluate the cancellous BMD by eliminating the confounding effect caused by osteosclerosis and osteophyte formation in the natural degenerative process [13, 49]. Given that regional differences in BMD exist in the cancellous bone of the vertebral body, studies have shown that HU values measured in the screw insertion position can better predict screw loosening risk [17, 18]. However, whether different regional BMD changes play different roles in screw anchorage ability has yet to be identified.

In this study, to verify this topic, comprehensive research consisting of clinical review and numerical mechanical simulations was performed. Clinical reviews present significantly better predictive performance of the pedicle region's HU value than other HU measurement methods when predicting screw loosening risk, and the biomechanical significance of pedicle BMD optimization has also been validated by fixation strength computation. Therefore, compared to other screw anchorage regions, the BMD of the pedicle plays a more important role in screw anchorage ability. Moreover, compared to the osteoporotic model, models with normal BMD in any position demonstrate better screw anchorage ability. Therefore, this study also confirms the potential biomechanical effects of anti‐osteoporosis therapy on optimizing screw anchorage ability from the biomechanical perspective [50, 51].

### 4.3 Mechanism Explanation and Clinical Significance

The current clinically observed and numerically computed results can be explained from the perspective of the load transmission pattern in pedicle screw fixed vertebral bodies. As mentioned above, screw compaction on bony structures caused by bone screw interface high stress is a main trigger for screw loosening [50, 52]. In the fixation system consisting of a pedicle screw and a connection rod, the main load was transported by the end of the screw [53, 54]. Correspondingly, compared to the screw tip and screw anchoraged to the vertebral body, a significantly higher stress value can be observed in the pedicle regional bony structures [53, 54]. Under this kind of biomechanical environment, BMD reduction (i.e., bony strength reduction) on the screw end with high stress could more significantly affect the screw anchorage ability adversely compared to other regions. In contrast, the reduction of BMD on the screw tip or screw–anchoraged vertebral body does not cause severe screw compaction on the bony structures for low stress in these regions. Therefore, the significance of these regional BMD reductions on the risk of screw loosening is limited compared to the pedicle region.

Recently, the adverse effect of BMD reduction on screw anchorage ability has been validated from several different perspectives [50, 54]. Correspondingly, the augmentation of the screw trajectory was shown to be an effective method to reduce the risk of screw loosening. However, limited by the contradiction on the biomechanical significance of different regional BMD on screw anchorage ability, surgeons have no definite conclusion on the selection of screw trajectory augmentation methods. The insertion of hydroxyapatite into the screw tip region was reported as an effective method to optimize the screw anchorage ability [20, 21], and cannulated pedicle screws with different bone cement distribution regions can also optimize the screw anchorage ability [55, 56]. However, the current study shows that the pedicle region, rather than the traditionally augmented screw tip or thread–augmented centrum regions, plays a more significant role in screw anchorage ability. Based on this study, the pedicle region augmentation strategy may be an alternative method to more effectively reduce the risk of screw loosening biomechanically compared to traditional screw trajectory augmentation strategies.

### 4.4 Methodology Selections

The identification of screw loosening risk by measuring HU values has been reported in published studies. In these studies, the HU value in screw loosening patients was significantly lower than that in patients without screw loosening. However, inconsistent with these studies, the HU values of the screw tip were insignificantly higher in patients suffering from left‐side screw loosening. This contradiction may be rooted in several cases with ideal BMD around the screw tip but severe osteoporosis on the pedicle region (Figure 3). Therefore, this phenomenon does not negate the reliability of the HU value in assessing vertebral body BMD and screw loosening risk; rather, it confirms the conclusions of this study from another perspective.

Traditionally, mechanical testing is the gold standard for screw anchorage ability judgment [57, 58]. However, we selected numerical mechanical simulations rather than mechanical tests to investigate changes in screw anchorage ability caused by BMD changes in different regions of screw trajectories. In addition to BMD changes, screw anchorage ability is affected by several factors, including screw orientations, pedicle sizes, and even outlines of vertebral bodies [59, 60]. When performing mechanical tests, it is difficult to unify these parameters. More importantly, the main aim of the study was to identify the biomechanical significance of different regions along screw trajectories, given that osteoporosis is a systemic disease, in the majority of specimens, in which BMD reduction occurs in all regions of the screw trajectory [7, 9]. Therefore, it is difficult to discretize the biomechanical significance of special region BMD changes.

In contrast, when computing the current research topic by a standard numerical model, all of the abovementioned covariates (screw orientations, pedicle sizes, and vertebral body outlines) are kept identical in different models. More significantly, we can precisely discretize special region BMD changes in numerical models, and the significance of different regional BMD changes can be accurately and directly reflected by the computational results of different models [17, 40]. Therefore, this study does not deny the necessity of mechanical tests, but numerical mechanical simulations can better meet the requirements of the current research topic. Moreover, the literature review indicates that the measurement of screw insertion torque can effectively predict screw anchorage ability [2, 61]. However, this study did not conduct a retrospective analysis. The relationship between insertion torque and the potential risk of screw loosening will be explored in our future research.

## 5 Strengths and Limitations

A clinical review, in conjunction with numerical mechanical simulation studies, offers new insights into the exploration of different screw trajectories. It underscores the critical role of BMD in the pedicle for effective screw anchorage and highlights its significant implications for screw augmentation strategies. Admittedly, the following limitations should be clarified in this study. First, only patients with L5 vertebral body screw loosening were enrolled in this study. This is because pedicle screw fixation is most commonly used in this vertebral body. Whether changes in motion segments will affect the current research topic should be verified. In addition, the biomechanical significance of the confounding factors (i.e., screw orientations, pedicle sizes, and interbody fusion status, etc.) and interactions between these parameters and screw trajectory BMD changes should also be verified in our future studies. Moreover, although radiography was still an effective method to judge screw loosening status, its sensitivity and specificity were still lower than those of this slice CT examination. Therefore, comprehensive research consisting of CT scan follow‐up patients and a more complex numerical model computation should be performed to revalidate the current research conclusion. Finally, it is widely acknowledged that bone cement augmentation serves as an effective method to enhance the anchorage capability of pedicle screws [50, 62]; however, this aspect is not directly related to the primary focus of our study. This topic has not been explored in the current research, and we intend to investigate cement distribution across various regions and its correlation with screw anchorage ability in our future studies.

## 6 Conclusion

Performing comprehensive research consisted of clinical review and numerical mechanical simulations. This study presents a significantly better predictive performance of the pedicle region HU value when predicting screw loosening risk and a more important biomechanical role of pedicle regional BMD changes. Therefore, the pedicle region augmentation strategy may be a more effective method to optimize the screw anchorage ability in osteoporotic patients.

## Author Contributions

**Zan Chen:** investigation, data curation, writing – review and editing. **Yue Chen:** conceptualization, supervision, project administration, funding acquisition. **Jiajun Zhou:** visualization, writing – review and editing. **Yanwei He:** writing – review and editing. **Jingchi Li:** methodology, validation, formal analysis, writing – original draft, writing – review and editing.

## Ethics Statement

Ethical approval for this study was obtained from the Ethics committee of the Affiliated Hospital of Southwest Medical University (KY2023291).

## Consent

The authors have nothing to report.

## Conflicts of Interest

The authors declare no conflicts of interest.

## Data Availability

All the data of the manuscript are presented in the paper.
